# Spatiotemporal effects on dengue incidence based on a large cluster randomized study

**DOI:** 10.1177/09622802251338371

**Published:** 2025-06-19

**Authors:** Jerome Johnson, Xiangyu Yu, Suzanne M Dufault, Nicholas P Jewell

**Affiliations:** 1 Institute of Clinical Trials and Methodology, MRC Clinical Trials Unit at University College London, London, UK; 2 Division of Biostatistics, School of Public Health, University of California, Berkeley, CA, USA; 3 Division of Biostatistics, Department of Epidemiology and Biostatistics, University of California, San Francisco, CA, USA; 4 Department of Medical Statistics, London School of Hygiene & Tropical Medicine, London, UK

**Keywords:** Cluster-randomized trial, cluster reallocation, dengue, disease clustering, spatiotemporal point process, spillover, test-negative studies

## Abstract

A recent large-scale cluster randomized test-negative study assessed the impact of a mosquito-based intervention on the incidence of clinical dengue showing a protective efficacy of 77.1% (95% CI: (65.3%, 84.9%)). While the intervention was randomized at a cluster-level, human and mosquito movement suggest potential violations in assumptions necessary for intention-to-treat analyses to produce accurate estimates of the full intervention effect due to spatial clustering of dengue cases, and/or potential non-independence in the intervention arising from spillover of the intervention (or control) across cluster boundaries. We address these distinct but related effects using two approaches. First, we examine whether a clustering effect exists, that is, whether the presence of a recent dengue case in the sample within a specified distance from a residence raises the risk of dengue. Second, we use cluster reallocation techniques to examine intervention spillover effects. We find strong spatial effects of the presence of dengue cases on the risk of clinical dengue that exhibit both serospecificity and a dose response, more evident in control than intervention clusters at least on an additive scale. Contrarily, there is no evidence of any appreciable local spillover effect from intervention to control clusters, or vice versa, in terms of either the risk of dengue infection or the level of disease clustering.

## 1. Introduction

The Applying *Wolbachia* to Eliminate Dengue (AWED) trial assessed the impact of a community-level intervention on the incidence of dengue in a large city, Yogyakarta, Indonesia, based on the geographic clusters.^1–3^ As is standard, clusters were randomized to either receive a community-level intervention or to act as control. Unlike traditional cluster-randomized trials which require the enrollment and intensive longitudinal surveillance of cluster cohorts, the study used test-negative sampling that exploited clinic-based surveillance systems to identify and enroll symptomatic health-care seeking patients. Once enrolled, individuals are tested for the disease of interest. Those who test positive are classified as cases, and those who test negative as controls. The process of sampling and enrolling individuals over the study period thus resembles a variant of a case-control study design superimposed over cluster randomization.

The primary intention-to-treat (ITT) analysis treated the intervention status of cluster of residence as an individual’s exposure. This approach exploited the cluster randomization scheme as the basis for inference, but did not consider the role of other risk factors for dengue incidence (although a constrained randomization approach was used that balanced arms on known risk factors for dengue including age and historical dengue incidence). Thus, spatial effects were not considered in the basic ITT analysis, nor the possibility that estimation of the intervention effect might be affected by spillover of either the intervention or control conditions across cluster boundaries. A further nuance arises since infection with dengue virus arises from four distinct serotypes where transmission is serotype-specific.^4^ That is, an individual infected with the serotype DENV1 cannot give rise in a future chain of transmission to infections other than those associated with serotype DENV1. To account for this, we explore the presence of global, serotype-specific and serotype-discordant disease clustering.

This work characterizes two consequences of the spatiotemporal natural of the intervention itself and the disease of interest: first, the risk of an individual acquiring a new dengue infection associated with the local presence of other dengue cases in the recent past, and second, potential spillover effects. We consider the impact of proximal dengue cases (in time and space) on an individual’s risk of dengue—evidence of disease clustering—with interest in determining whether the relationship differs between intervention and control clusters. This can be achieved by defining a new proximity risk factor and incorporating it into basic logistic regression models that capture intervention efficacy while accounting for this clustering. For spillover, we consider a sensitivity analysis based on cluster reallocation schemes which enlarge or shrink cluster boundaries. Both analyses build on recent work, including a report on the spatiotemporal clustering of dengue that used a different approach, providing insight into the focal transmission of dengue in the intervention and control areas of the AWED study^5^ as well as a spatiotemporally resolved reanalysis of the AWED data, providing evidence of potential underestimation of the intervention effect due to human and mosquito movement within the AWED study.^6^

## 2. Effect of a proximal dengue case

**Figure 1.**
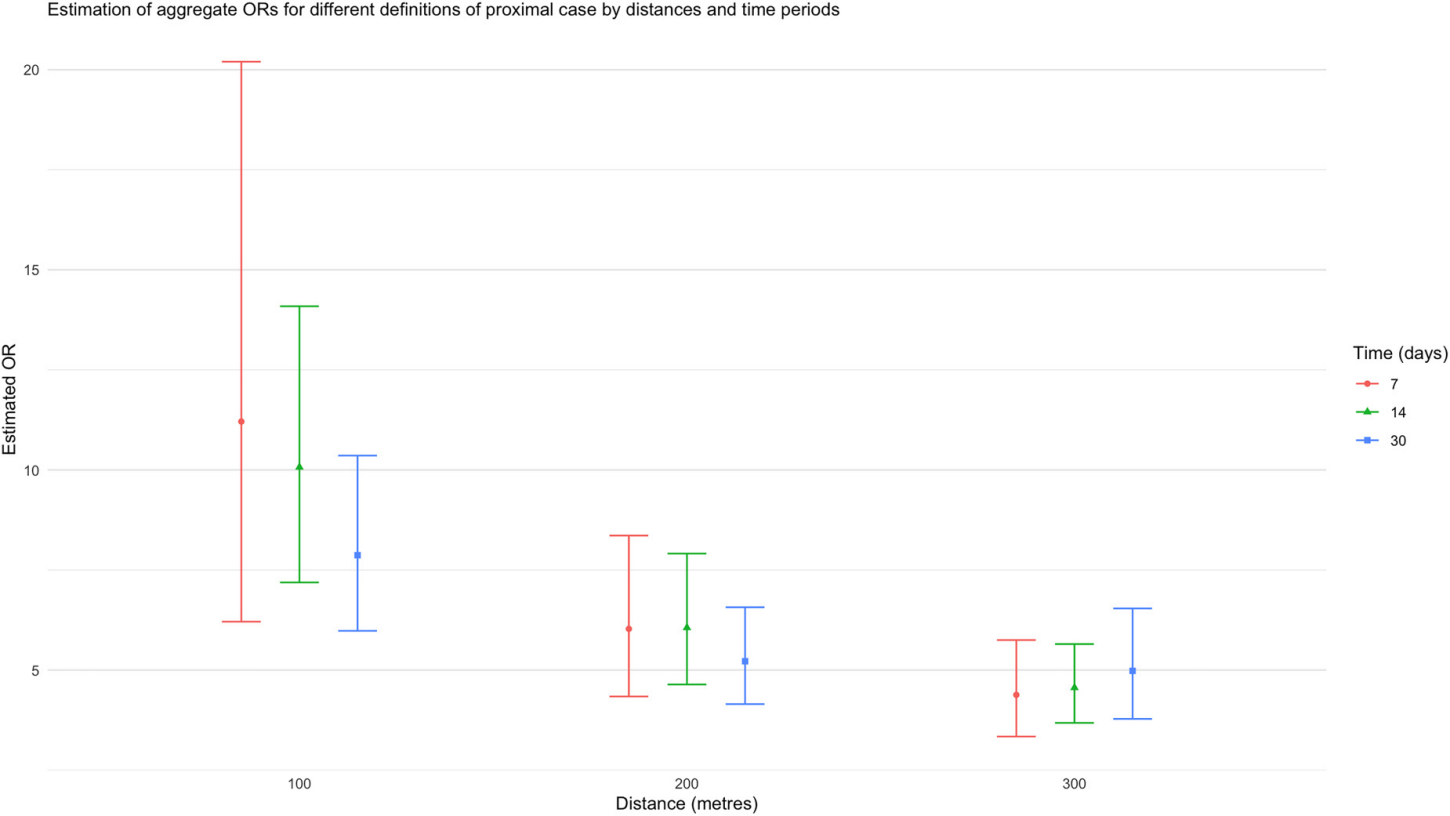
The estimated aggregate odds ratio (OR) associated with a proximal case, when the definition of “proximal” changes in space and time.

We first examine the spatial impact of recent prevalent dengue cases on the risk of an individual acquiring a new dengue infection. To explore such effects we use geographic information system (GIS) information to locate all dengue cases in the study and their proximity in both space and time to all other participants, allowing us to create a proximal case indicator. Ignoring serotype, Table 1 provides a classification of dengue cases and test-negatives by whether the residence of any individual was located “close” to a dengue case in the data set, specifically a dengue case that had occurred in the prior 30 days for a participant whose residence was also within 300 m of the individual’s home location, these distances being based on the earlier work.^5^ This indicator variable thereby acts as a proxy for close prevailing dengue infections. Note that this table includes 67 dengue cases for whom the serotype was unknown. Seven individuals were infected by two different dengue serotypes at different points of time and only the first of these infections are included here. The estimated odds ratio (OR) associated with the presence of a proximal case, is 4.98 (95% CI: (3.78, 6.54)), reflecting the impact of local spread of infection (Figure 1); estimation and inference is carried out according to Jewell et al.^3^; here, variability of estimation of the OR is first measured on the log scale and estimated using the standard robust sandwich estimator that accounts for clustering. This OR does not change materially ( $\hat{OR}$ = 4.65, 95% CI: (3.50, 6.18)) when the 67 unknown serotypes are removed from the analysis. As illustrated in Figure 1, the estimated OR increases when either the time period is reduced from 30 days or the distance is reduced from 300 m, although the uncertainty increases also due to smaller number of exposed when the proximal measure is tightened. For example, if a proximal case is defined as within 7 days and with a residence within 100 m, the estimated OR is 11.2, (95% CI: (6.21, 20.1)). Recall that these ORs reflect estimates of the relative risk ( RR ) of being a test-positive dengue case comparing participants with recent proximal exposure (as defined explicitly) as compared to those without such exposure.^3^ From this, the efficacy of the intervention is immediately determined by 100×(1−RR) .

**Table 1.** Effects of intervention and exposure on risk of a DENV, where a participant’s exposure is defined by a dengue infection occurrence (of another participant) within the prior 30 days and with a home location within 300 m of the infected participant. The ORs reported in the bottom half of the table are based on a logistic regression model, using GEEs to account for clustering, with CIs based on the standard sandwich variance estimator.

Variable	OR	(95% CI)	*p*-value	
Exposure	4.08	(3.06, 5.46)	0.00	<0.001
Intervention	0.33	(0.23, 0.49)	0.00	<0.001
Exposure × Intervention	0.67	(0.38, 1.18)	0.16	

DDENV: test-positive dengue case; GEEs: generalized estimating equations; ORs: odds ratios; CI: confidence interval.

The estimated ORs associated with a proximal case (with a space-time window of 300 m and 30 days) are 2.72 (95% CI: (1.66, 4.46)), and 4.08 (95% CI: (3.06, 5.46)) in the intervention and control arms, respectively. Note that a participant’s intervention arm is determined by their place of residence but a proximal case may not be in the same arm. Although this difference does not provide strong evidence that the effect of a recent proximal dengue case differs (multiplicatively) between treatment and control clusters (the *p*-value associated with the interaction term is 0.16), it nevertheless suggests more disease clustering in control clusters than in intervention clusters; further, the findings suggest a degree of *additive* interaction induced by the greater frequencies of dengue infections in control, as compared to intervention, clusters.

Note that, the reported marginal intervention OR for the trial is 0.23 (95% CI: (0.15, 0.35)),^1^ when there is no adjustment for the existence of proximal cases. The data in Table 1 yield an estimated intervention OR of 0.33 (95% CI: (0.23, 0.49)) when there is no proximal case; when a proximal case is present the intervention OR decreases (and thus efficacy increases) slightly to 0.22 (95% CI: (0.11. 0,46)), although this small change is not quite statistically significant (*p* = 0.16). When the interaction term is omitted, the estimated intervention OR is 0.30 (95% CI: (0.20, 0.46)), reported in Tables 2 to 7. This slight increase in the OR when proximal cases are included as a risk factor (as compared to the original ITT estimate) does not reflect any diminution of the efficacy of the intervention, but suggests that a small part of the success of the intervention in reducing risk is accounted for by a reduction in the number of proximal cases in intervention clusters. In this sense, the proximal risk factor is acting as a mediator of a small component of the intervention effect, so that the OR of 0.30 can then be interpreted as a measure of the direct effect of the intervention.

**Table 2.** Effects of intervention and proximal exposures on the risk of infection with dengue of any serotype, where proximal exposure is defined by existence of an infection occurrence within the past 30 days with a home location within 300 m; exposure dose captures the number of proximal DENV cases according to this same definition. The ORs are based on a logistic regression model (without interaction), using GEE to account for clustering, with CIs based on the standard sandwich variance estimator.

Variable	OR	(95% CI)	*p*-value	
Exposure	3.86	(2.98, 5.01)	0.00	<0.001
Intervention	0.300	(0.198, 0.457)	0.00	<0.001
Variable	OR	(95% CI)	*p*-value	
Dose exposure	1.56	(1.36, 1.79)	0.00	<0.001
Intervention	0.294	(0.197, 0.439)	0.00	<0.001

DENV: test-positive dengue case; GEEs: generalized estimating equations; ORs: odds ratios; CI: confidence interval.

**Table 3.** Effects of intervention and exposure on infection with dengue serotype DENV1, where a participant’s exposure is defined by a dengue infection occurrence (of another participant) within the prior 30 days and with a home location within 300 m of the infected participant.

		Aggregate			Intervention			Control		
Serotype	Exposure	DENV1	Test negative	OR (CI)	DENV1	Test negative	OR (CI)	DENV1	Test negative	OR (CI)
DENV1	Exposed	11	140	9.87	0	38	0.00	11	102	9.46
	Unexposed	46	5781	(2.70, 36.1)	12	2800	–	34	2981	(2.64, 33.9)
DENV2	Exposed	12	515	2.80	2	122	4.45	10	393	1.96
	Unexposed	45	5406	(1.54, 5.09)	10	2716	(1.37, 14.4)	35	2690	(1.04, 3.66)
DENV3	Exposed	1	113	0.92	1	14	18.3	0	99	0.00
	Unexposed	56	5808	(0.10, 8.16)	11	2824	(3.58, 94.0)	45	2984	–
DENV4	Exposed	6	301	2.20	0	83	0.00	6	218	2.02
	Unexposed	51	5620	(0.86, 5.62)	12	2755	–	39	2865	(0.78, 5.23)

DENV: test-positive dengue case; ORs: odds ratios; CI: confidence interval.

**Table 4.** Effects of intervention and exposure on infection with dengue serotype DENV2, where a participant’s exposure is defined by a dengue infection occurrence (of another participant) within the prior 30 days and with a home location within 300 m of the infected participant.

		Aggregate			Intervention			Control		
Serotype	Exposure	DENV2	Test negative	OR (CI)	DENV2	Test negative	OR (CI)	DENV2	Test negative	OR (CI)
DENV1	Exposed	26	140	8.39	2	38	8.19	24	102	6.38
	Unexposed	128	5781	(5.44, 12.9)	18	2800	(2.99, 22.4)	110	2981	(4.22, 9.63)
DENV2	Exposed	73	515	9.46	0	122	0.00	73	393	8.19
	Unexposed	81	5406	(7.06, 12.7)	20	2716	–	61	2690	(6.47, 10.4)
DENV3	Exposed	2	113	0.68	0	14	0.00	2	99	0.46
	Unexposed	152	5808	(0.16, 2.91)	20	2824	–	132	2984	(0.10, 2.02)
DENV4	Exposed	19	301	2.63	1	83	1.75	18	218	2.04
	Unexposed	135	5620	(1.37, 5.05)	19	2755	(0.14, 2.20)	116	2865	(1.12, 3.73)

DENV: test-positive dengue case; ORs: odds ratios; CI: confidence interval.

**Table 5.** Effects of intervention and exposure on infection with dengue serotype DENV3, where a participant’s exposure is defined by a dengue infection occurrence (of another participant) within the prior 30 days and with a home location within 300 m of the infected participant.

		Aggregate			Intervention			Control		
Serotype	Exposure	DENV3	Test negative	OR (CI)	DENV3	Test negative	OR (CI)	DENV3	Test negative	OR (CI)
DENV1	Exposed	1	140	1.59	1	38	18.4	0	102	0.00
	Unexposed	26	5781	(0.20, 12.9)	4	2800	(2.12, 109)	22	2981	–
DENV2	Exposed	2	515	0.84	0	12	0.00	2	393	0.68
	Unexposed	25	5406	(0.30, 2.38)	5	2716	–	20	2690	(0.24, 1.95)
DENV3	Exposed	9	113	25.7	3	14	303	6	99	11.3
	Unexposed	18	5808	(6.49, 102)	2	2824	(52.7, 1740)	16	2984	(2.51, 50.9)
DENV4	Exposed	3	301	2.33	0	83	0.00	3	218	2.08
	Unexposed	24	5620	(0.54, 10.1)	5	2755	–	19	2865	(0.46, 9.27)

DENV: test-positive dengue case; ORs: odds ratios; CI: confidence interval.

**Table 6.** Effects of intervention and exposure on infection with dengue serotype 4 (DENV4), where a participant’s exposure is defined by a dengue infection occurrence (of another participant) within the prior 30 days and with a home location within 300 m of the infected participant.

		Aggregate			Intervention			Control		
Serotype	Exposure	DENV4	Test negative	OR (CI)	DENV4	Test negative	OR (CI)	DENV4	Test negative	OR (CI)
DENV1	Exposed	11	140	5.98	0	38	0.00	11	102	5.45
	Unexposed	76	5781	(2.98, 12.0)	17	2800	–	59	2981	(2.93, 10.1)
DENV2	Exposed	19	515	2.93	3	122	4.77	16	393	2.03
	Unexposed	68	5406	(1.62, 5.30)	14	2716	(2.15, 10.6)	54	2690	(1.08, 3.81)
DENV3	Exposed	5	113	3.13	1	14	12.6	4	99	1.83
	Unexposed	82	5808	(1.14, 8.60)	16	2824	(2.26, 70.3)	66	2984	(0.66, 5.07)
DENV4	Exposed	22	301	6.32	1	83	2.07	21	218	5.63
	Unexposed	65	5620	(4.57, 8.74)	16	2755	(0.42, 10.2)	49	2865	(3.94, 8.06)

DENV: test-positive dengue case; ORs: odds ratios; CI: confidence interval.

**Table 7.** Effects of intervention and all serotype proximal exposures, where exposures are defined by existence of an infection occurrence within the past 30 days with a home location within 300 m; outcomes are infections by different serotypes

	OR of DENV1	OR of DENV2	OR of DENV3	OR of DENV4	OR of
Outcome	exposure	exposure	exposure	exposure	Intervention
DENV1 infection	7.07	1.66	0.63	1.29	0.35
(95% CI)	(1.82, 27.5)	(0.77, 3.60)	(0.07, 5.85)	(0.56, 2.97)	(0.14, 0.89)
*p*-value	<0.001	0.20	0.68	0.55	0.03
DENV2 infection	3.96	6.06	0.41	1.07	0.24
(95% CI)	(2.42, 6.47)	(4.57, 8.03)	(0.12, 1.41)	(0.59, 1.95)	(0.15, 0.38)
*p*-value	<0.001	<0.001	0.16	0.82	<0.001
DENV3 infection	1.39	0.53	19.4	1.17	0.34
(95% CI)	(0.17, 11.4)	(0.17, 1.62)	(4.83, 78.1)	(0.48, 2.85)	(0.09, 1.27)
*p*-value	0.76	0.27	<0.001	0.73	0.11
DENV4 infection	3.66	1.47	1.76	4.13	0.34
(95% CI)	(2.00, 6.69)	(0.79, 2.74)	(0.70, 4.41)	(2.80, 6.09)	(0.22, 0.54)
*p*-value	<0.001	0.23	0.23	<0.001	<0.001

DENV: test-positive dengue case; ORs: odds ratios; CI: confidence interval.

### 2.1. Serotype specific analysis

To perform a more nuanced analysis of linked transmission cases, we considered only homotypic dengue cases, that is where the serotype of the outcome dengue case matches the serotype of a proximal case. Tables 3 to 6 provide a classification of dengue cases, for each of the four distinct serotypes, and test-negatives (controls), by whether the participant was exposed to a recent (e.g. within the last 30 days) dengue case of the *matching* serotype within a specified distance (here, 300 m, again). Dengue cases with unknown serotype are excluded from the analysis; however, the seven individuals infected twice with different serotypes are included in the analyses corresponding to each of their serotypes. The data is again sub-divided by whether the subjects lived in an intervention or control cluster. Tables 3 to 6 provide aggregate ORs, capturing the impact of the existence of a local recent homotypic case (that matches the serotype of the specific outcome variable), together with 95% confidence intervals based on the sandwich estimates of the standard errors of the OR estimate on the log scale.

For example, Table 3 shows that the presence of a proximal DENV1 dengue case yields an estimated exposure OR, of being a test-positive (specifically, also DENV1) as compared to a test-negative or other dengue serotype, of 9.87 (95% CI: (2.70, 36.1)). Similarly, for DENV2–DENV4, the analogous estimated ORs are 9.46 (95% CI: (7.06, 12.7)), 25.7 (95% CI: (6.49, 102)), and 6.32 (95% CI: (4.57, 8.74)), respectively. All of these effects are large and statistically significant, but with some uncertainty due to small number of exposed cases. Again, these ORs reflect estimates of the relative risk of a test-positive dengue case of a given serotype, comparing participants with recent proximal exposure to the relevant serotype to those without such exposure (and subsequently the intervention efficacy).

At the first glance, the effects of a recent, proximal dengue case on risk do not appear, however, to be entirely specific to the recent case being of the same serotype, but tend to be smaller for a non-matching serotypic proximal case, at least for these univariate analyses. For example, for DENV1 cases, the estimated OR for a recent close case of DENV2, DENV3, and DENV4, are 2.80 (95% CI: (1.54. 5.09)), 0.92 (95% CI: (0.10, 8.16)), and 2.20 (95% CI: (0.86, 5.62)), respectively. Only one of these effects achieves statistical significance. It is worth noting that DENV3 was the serotype with the least observed cases overall.

Similar patterns are observed for other serotypes in Tables 4 to 6. It is possible that the presence of a single case of dengue in a local area reflects an increased prevalence of several, or all, dengue serotypes in the mosquito population at that time, explaining some deviations from serospecificity. Further, single serotype case numbers are sufficiently small (particularly in intervention clusters) to make it impossible to detect any patterns of (multiplicative) interaction across intervention and control clusters at this level of detail.

The serotype-specific analyses of Tables 3 to 6 were carried out separately for each possibility of a close dengue serotype without controlling for the presence of cases of other serotypes. As noted, it is thus possible that the risk of a DENV1 infection associated with the close presence of a dengue DENV2 individual may simply reflect the correlated presence of other undetected individuals with dengue DENV1 at the same time. A multivariate logistic regression model can examine this issue as well as the underlying main effect of the intervention which is masked in Tables 3 to 6. Table 7 provides the result of such analyses for each serotype outcome in turn.

When accounting for the existence of proximal cases of each serotype, Table 7 shows that the patterns of estimated ORs are similar to the marginal analyses shown in Tables 3 to 6. For example, when the outcome is infection with DENV1, the estimated OR (and 95% CI) associated with the presence of a proximal dengue case of DENV1, DENV2, DENV3, and DENV4 are 7.07 (1.82, 27.5), 1.66 (0.77, 3.60), 0.63 (0.07, 5.85), and 4.13 (0.56, 2.97), respectively. Thus, although this multiple regression analysis slightly mutes the estimated OR from those shown in Table 3, the pattern of associations remains unchanged. However, none of the ORs for proximal cases of DENV2, DENV3, or DENV4 are significantly different from the null value, 1, when the outcome is infection with DENV1, and fewer significant non-serotype specific associations are observed than in the univariate analyses. In this sense, the proximal case analysis shows serospecificity, validating the effect of proximal exposure measurement.

Table 2 re-examines the data from Table 1 without including an interaction effect between intervention and proximal exposure (and ignoring serotype). We commented above on the interpretation of the intervention effect described here as compared to the ITT analysis. Note that, ignoring interaction, the estimated OR associated with the presence of any proximal case (within 300 m and 30 days) is 3.86 with a 95% CI (2.98, 5.01).

In addition, Table 2 considers a dose response effect underlying the data in Table 1; that is, when exposure is measured by the *number* of proximal cases of dengue infection. The range of such counts in the data is from 0 to 4 for the space-time window of 300 m and 30 days, respectively. The estimated OR associated with an increase of one proximal case is 1.56, reflecting a notable dose response, to some extent reflecting that proximal case measures are picking up the prevalence level of dengue in the local vicinity with higher levels increasing the risk of infection as would be expected. This further validates the proximal case measurement to capture infection risk.

Concluding this section, there is a noticeable and large effect of the existence of a proximal dengue infection on a participant’s risk of dengue infection. The intervention effect estimate does not materially change by accounting for this phenomenon. This proximal effect is not modified (multiplicatively) when comparing intervention and control clusters. However, the absence of multiplicative interaction (with such large proximal and intervention main effects) indicates considerable *additive* interaction where the risk difference (or excess risk) must be much larger in the control clusters than in intervention clusters. The existence of such additive interaction suggests a causal effect of the intervention on the impact of proximal dengue infections.^7^ In other words, there is not only reduced dengue infections in intervention clusters, there is also less clustering of dengue cases also. This supports findings of Dufault et al.^5^ who used a spatial clustering measure to arrive at the same conclusion. We discuss potential spillover effects on both approaches in the next section. We could also attack this question in a similar fashion by looking at buffered estimates of the proximal case effects captured in Section 2 but have not pursued this further here in the interest of space.

## 3. Cluster reallocation and spillover effects

### 3.1. Spillover effects

Spillover effects are the indirect effect of an intervention on individuals who are in physical or social proximity to other intervention recipients.^8^ The difference between spillover effects and the more commonly used term of contamination is that contamination involves individuals unintentionally receiving or being exposed to the intervention whereas spillover affects the individuals who do not receive the intervention but are affected by those who do in close proximity; that is, it can occur without individuals in the control arm directly receiving the intervention. Note that the converse also holds true for spillover effects as it can also refer to the outcomes of individuals receiving the intervention being affected by those in close proximity who do not. In cluster randomized trials based on the location, there is often an assumption of the absence of between-cluster spillover in that people and diseases are assumed to move freely within a cluster but are not affected by events in other clusters. In the AWED trial, it is evident that there is potential for both spillover and contamination effects since clusters border each other as compared to being in separate geographical locations such as schools in a city. Thus, the assumption that there are no between-cluster effects (both spillover and contamination) is open for question in this context.

Spillover effects can also be split into positive or negative effects. A positive spillover effect benefits individuals who are affected by the spillover, and a negative spillover effect harms individuals who are affected by the spillover. In the AWED setting, it is possible that, if fewer infections occur in intervention clusters, control individuals living close to the border of an intervention cluster may experience less risk of infection as a result of a positive spillover effect. Alternatively, large numbers of infections in a control cluster may increase the infection risk in those areas of intervention clusters close to the boundary with a control cluster.

Contamination is also possible because of migration of mosquitos across cluster boundaries or, more importantly, through human mobility whereby some individuals spend significant time in clusters other than where they live.^6^ Human mobility patterns suggest that such contamination can occur far from cluster boundaries. Here we focus on detection of spillover (and local contamination) effects that occur close to cluster boundaries. Fogelson et al.^9^ consider accounting for general contamination effects, as captured by measurements on both mosquito and human mobility, using a constructed exposure index but do not assess potential spillover.

Valid inference in randomized experiments rely on the stable unit treatment value assumption which requires that differences between the outcomes of control and intervention participants only depend on the participant’s intervention status and not on what intervention others receive—sometimes referred to as non-interference. Spillover effects violate this assumption, even at the cluster level, resulting in potential bias of causal effects estimated from randomized comparisons. The identification of possible local spillover effects in cluster randomized trials is valuable in order to attempt to take them into account when assessing causal comparisons; even though it adds complexity, the knowledge of the existence and the direction of spillover effects may also help in the implementation of intervention strategies in the future.

There is currently no standard statistical method of identifying the existence of spatial spillover, although some ad hoc approaches have been utilized in the analysis of cluster randomized trials.^8^

### 3.2. Cluster reallocation

Jarvis proposed a method, termed “cluster reallocation,” that explores the presence of spatial spillover in cluster randomized trials.^8^ Other than assuming that spillover is based on the proximity to cluster boundaries, this method makes no further assumptions about the spillover mechanism.

Cluster reallocation is implemented by reassigning participants to intervention or control arms depending on their proximity to cluster boundaries. The general idea is that the intervention cluster boundaries are “buffered” by a pre-determined distance, for example, 50 m, so that the participants in the control arm that are within this 50 m buffer of the intervention boundary are now reassigned (or reallocated) into the intervention arm; the estimand of interest is then recalculated using the newly defined trial arms. In essence, the intervention clusters are expanded geographically slightly, simultaneously shrinking the control clusters.

The reallocation process is then repeated for incrementally increased buffer distances. This process can also be repeated in the same manner for the control cluster boundaries meaning that control clusters are now expanded by some given distance with participants in the intervention arm that are within this control boundary buffer reallocated as control participants. Again, the estimand is recalculated, at various control buffer distances. The method provides estimates of the intervention effect for hypothetical spatial definitions of the intervention and control arms, known as “buffered estimates.”^8^

When spillover is absent, increases in the intervention or control clusters boundaries result in weaker estimated intervention effects because of misclassification. It is hypothesized that in the absence of spillover, the observed study estimate will be either the maximum or minimum estimate (depending on the estimand of interest) compared to buffered estimates because reallocation of observations to different trial arms will tend to increase similarities between the two arms thereby diluting the intervention effect, and so forth.

### 3.3. Applications to AWED data

We consider the application of cluster reallocation to two estimands of interest in the AWED trial: (i) the intervention effect (controlling for the presence of local recent cases) as discussed in Section 2, and (ii) a spatial measure of disease clustering discussed by Dufault et al.^5^

For any selected buffer, a binary vector is defined that returns a value of 1 if participants are within buffered intervention clusters and 0 otherwise. This binary vector is then added to the merged data set as the “new” treatment variable since it defines the reallocated intervention and untreated observations. The choices of buffer distances considered is based on the earlier work and analyses of Dufault et al.^5^

**Figure 2.**
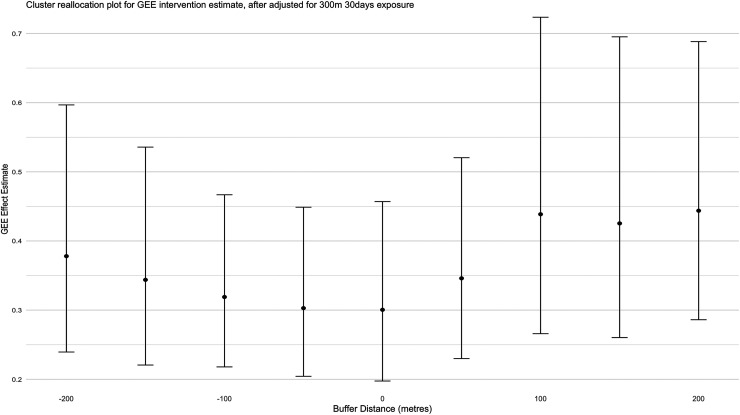
Intervention odds ratio (after adjustment for presence of proximal cases within 30 days and 300 m) for various buffer values for cluster reallocation. Error bars indicate the pointwise 95% confidence intervals estimated using a standard robust sandwich estimator.^3^

**Figure 3.**
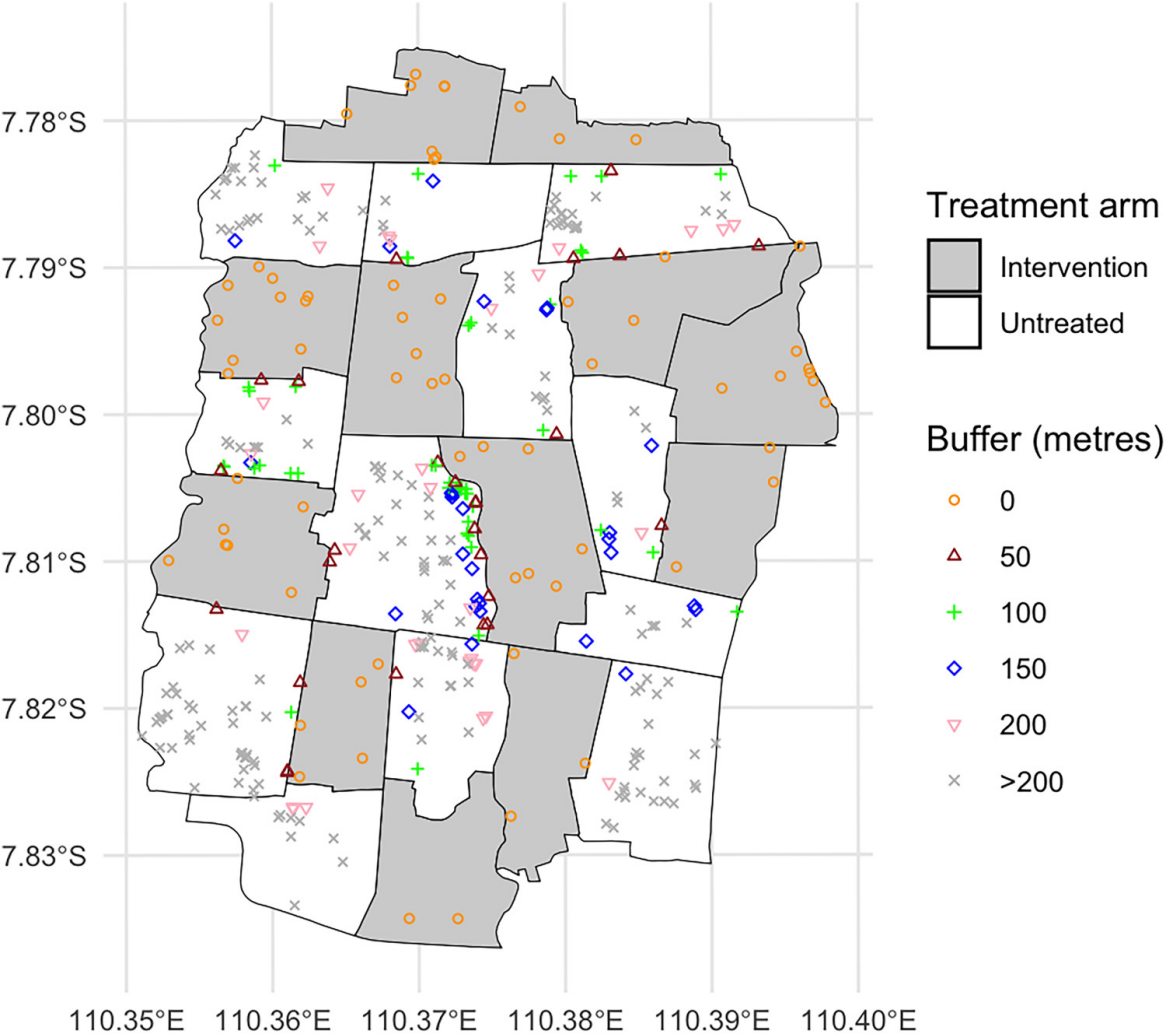
Residential locations of the dengue test-positive cases across the Applying Wolbachia to Eliminate Dengue (AWED) study area. Different shapes indicate which cases fall within the “new” intervention region as the buffer distances increase from 0 to + 200 m. Dengue test-positive cases that remain in the “untreated” region at distances >200 m from an intervention area boundary are visualized as gray “x”s.

In the first case, Figure 2 shows the intervention odds ratio (adjusting for the presence of a proximal infection—within 300 m and during the prior 30 days, as in Section 2) for differing buffer distances up until ±200 m. A maximum buffer distance of 200 m was chosen since each cluster is ∼ 1 km ${}^{2}$ in area, and greater buffer distances resulted in small number of participants in some clusters (Figure 3). For simplicity, this figure only demonstrates the effect on the “new” intervention assignments for cases within increasing buffer distances around the intervention region (+0 to +200 m), though a similar figure could also be generated to visualize decreasing buffer distances. Reading from Figure 2, for example, the estimated intervention OR with a buffer zone of +200 m increases to 0.44 (from 0.30). That is, we see a diluted intervention effect when we expand intervention clusters by 200 m throughout. This occurs for all buffer distances shown including when intervention clusters are shrunk, suggesting that there is little evidence of spillover or local contamination in terms of measuring an intervention effect.

The second application focuses on an alternative measure of spatial clustering. To assess small-scale spatiotemporal clustering in dengue cases throughout the trial area, we consider a global measure of spatiotemporal clustering, τ .^10^ This measure captures the overall tendency of homotypic dengue cases (i.e. cases of the same serotype) to occur within specified space-time windows *above and beyond* that observed in the enrolled study population due to secular factors such as healthcare-seeking behavior and environmental conditions. The numerator of the ratio-based estimator, τ identifies, among those enrolled within a particular space-time window, the number of serotype-homotypic dengue pairs relative to the number of pairs of enrolled individuals who are assumed not to be transmission-related, the latter group including the test-negative controls as well as any heterotypic dengue pairs. As such, the numerator of the τ -statistic reflects an estimate of the odds of observing a homotypic dengue pair among all enrolled pairs in a given space-time window. The denominator of the τ -statistic is constructed the same way, but without restriction on the spatial window. Therefore, τ>1 indicates that two participants are more likely to be homotypic dengue cases if they fall within the specified space-time window than if they fall anywhere across the study area.^5^ Note that cases with unknown serotype are removed for these calculations. Further, since Dufault et al. noted differing clustering of dengue cases in the intervention and control arms,^5^ we compute τ separately for the two treatment arms. The calculations for the control arm can be found in the Supplemental Material.

Using the same reallocated binary vector as above for each buffer, the τ statistic, as defined above, was calculated for the intervention arm using the new definitions of intervention and control observations. The space–time window of 300 m and 30 days was used in the definition of τ in all cases. A 95% CI was also obtained through the use of a permutation-based null distribution^5^ (where null here means no spatial clustering), with 1000 iterations each time, based on the reallocated observations, in order to identify if spatial dependence is detected in the reallocated clusters.

**Figure 4.**
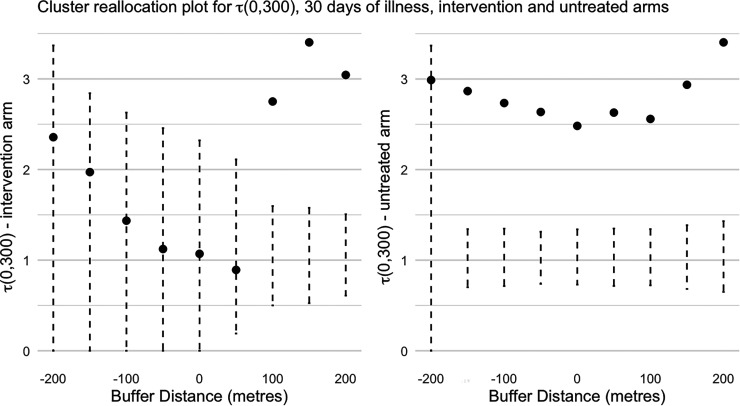
The spatial clustering measure, τ , (with a space-time window of 300 m and 30 days) in intervention and untreated clusters in the Applying Wolbachia to Eliminate Dengue (AWED) trial, after cluster reallocation at various buffer distances. Error bars indicate the pointwise 95% confidence interval from the permutation-based null rejection region based on 1000 permutations of the data.^5^

This process was repeated by modifying the buffer distance by 50 m in each direction consecutively up until 200 m, as before. This process was also repeated in an analogous way in the control clusters. The results for both arms are shown in Figure 4. According to Dufault et al.,^5^ with no cluster reallocation (i.e. a zero buffer), there is no evidence of disease clustering in the intervention arm as the point estimate of τ lies squarely in the middle of the 95% null permutation-based CI. In other words, the level of observed disease clustering is entirely compatible with the premise of no disease clustering. This is in contrast to the control arm where there is substantial evidence of disease clustering,^5^ suggesting that the intervention not only reduces dengue incidence but also disrupts patterns of local disease transmission (i.e. clustering). In other words, sporadic cases tend not to generate disease clusters in the intervention arm where there is strong evidence that they do in the control arm.

We now turn to the cluster reallocation results for τ , as shown in Figure 4, with both a positive and negative buffer. Although the size of the estimated τ increases as we shrink the intervention clusters (i.e. a negative buffer), so does the level of uncertainty due to shrinking sample sizes with smaller intervention clusters, so that the interpretation remains the same that there is no evidence of disease clustering. However, when we expand the intervention clusters (i.e. using positive buffers), strong evidence of disease clustering appears as soon as the buffer is large as only 100 m. This reflects that some disease clustering in the control arm is present close to cluster boundaries and contaminates the τ metric as soon as these control cluster participants are reallocated to the intervention arm. This suggests that there is no spillover effect from the intervention clusters to the control clusters in terms of disease clustering, or at least that the spillover only impacts participants who live <100 m from a cluster boundary.

## 4. Discussion

By exploiting GIS information on participants’ home residence locations in the AWED trial, we have demonstrated a sharp increase in risk of dengue incidence associated with the local presence of other dengue cases in the recent past. This effect occurs in both arms but much more strongly (on the additive scale) in control clusters, suggesting increased disease clustering for control individuals. This is confirmed through use of a spatial clustering metric, τ , demonstrating the disruption of local transmission patterns by the intervention. There is little evidence of local spillover, or contamination, between clusters in either a positive or negative direction, either as measured by intervention efficacy or by a spatial clustering metric.

The serospecific analyses of Section 2 are carried out separately, by turn, for each of the four dengue serotypes (e.g. in Table 7). Since these analyses are necessarily correlated (each analysis uses the same test-negative controls), one would ideally like to implement a single multinomial logistic regression analysis that accommodates each serotypic outcome simultaneously. While this is straightforward in principle if we include the four proximal case risk variables together for each of the four mutinomial outcomes, it requires a model with a large number of unknown multinomial logistic regression coefficient parameters (particularly if we allow the risk relationships to differ between intervention and control clusters). The small number of cases makes such a model problematic for the AWED trial data. Furthermore, we are unaware of any multinomial logistic regression software that allows different risk variables for predicting the separate serotypic disease outcomes that would be necessary to fit a more parsimonious serospecific model. A submodel of this latter type would be useful to globally assess the evidence that proximal risk variables for a specific serotype only predict the matching serotype incidence outcome.

The AWED trial spatial data represent an example of a spatiotemporal point process, suggesting consideration of a model for the conditional infection intensity at a given location, x , and time t , given the history of the process up to time t . Predictors of the intensity at location x and time t might depend on a kernel function that measures both geographic and temporal distance from past observations. Models of this kind are extensions of survival analysis regression models such as the Cox proportional hazards model, and have been implemented for other infectious disease applications including foot and mouth disease outbreaks in cattle,^11^ the latter analysis based on a partial likelihood approach. The case-cohort analyses of Section 2 are essentially special cases of this method using a very simple indicator kernel function.

Most observed spatiotemporal point processes suffer from only observing spatiotemporal information on cases. The test-negative design has a significant advantage in that the method naturally provides data to study spatiotemporal incidence functions more effectively as test-negative participants provide natural comparative spatiotemporal control information.

Finally, the lack of evidence of spillover or local contamination of the intervention is an important finding for the design of future cluster randomized vector control studies in an urban setting where clusters are necessarily adjacent.

## Supplemental Material

sj-pdf-1-smm-10.1177_09622802251338371 - Supplemental material for Spatiotemporal effects on dengue incidence based on a large cluster randomized study Supplemental material, sj-pdf-1-smm-10.1177_09622802251338371 for Spatiotemporal effects on dengue incidence based on a large cluster randomized study by Jerome Johnson, Xiangyu Yu, Suzanne M Dufault and Nicholas P Jewell in Statistical Methods in Medical Research
