# Supplementary material for: Spatiotemporal effects on dengue incidence based on a large cluster randomized study
Source: Stat Methods Med Res. 2025 Jun 19;34(7):1303–13. doi: 10.1177/09622802251338371 (PMC12308035; doi:10.1177/09622802251338371)
Supplement: sj-pdf-1-smm-10.1177_09622802251338371 - Supplemental material for Spatiotemporal effects on dengue incidence based on a large cluster randomized study [file sj-pdf-1-smm-10.1177_09622802251338371.pdf]

Spatio-Temporal Effects on Dengue Risk Based on a Large Cluster Randomised Study

Supplemental Material

JEROME JOHNSON+
Institute of Clinical Trials and Methodology
MRC Clinical Trials Unit at University College London
London, WC1V 6BH, United Kingdom

XIANGYU YU+
Division of Biostatistics
School of Public Health
University of California, Berkeley, CA 94720, USA

SUZANNE M. DUFAULT*
Division of Biostatistics
Department of Epidemiology and Biostatistics
University of California, San Francisco, CA 94158, USA

NICHOLAS P. JEWELL
Department of Medical Statistics
London School of Hygiene & Tropical Medicine
London, WC1E 7HT, United Kingdom

+ Jerome Johnson and Xiangyu Yu are considered co-first authors of this manuscript.

May 15, 2024

*Send correspondence to: Suzanne M. Dufault, Department of Epidemiology and Biostatistics, University of California at San Francisco, Mission Hall: Global Health and Clinical Sciences, Box 0560, 550 16th St, San Francisco, CA 94143, USA E-mail: suzanne.dufault@ucsf.edu

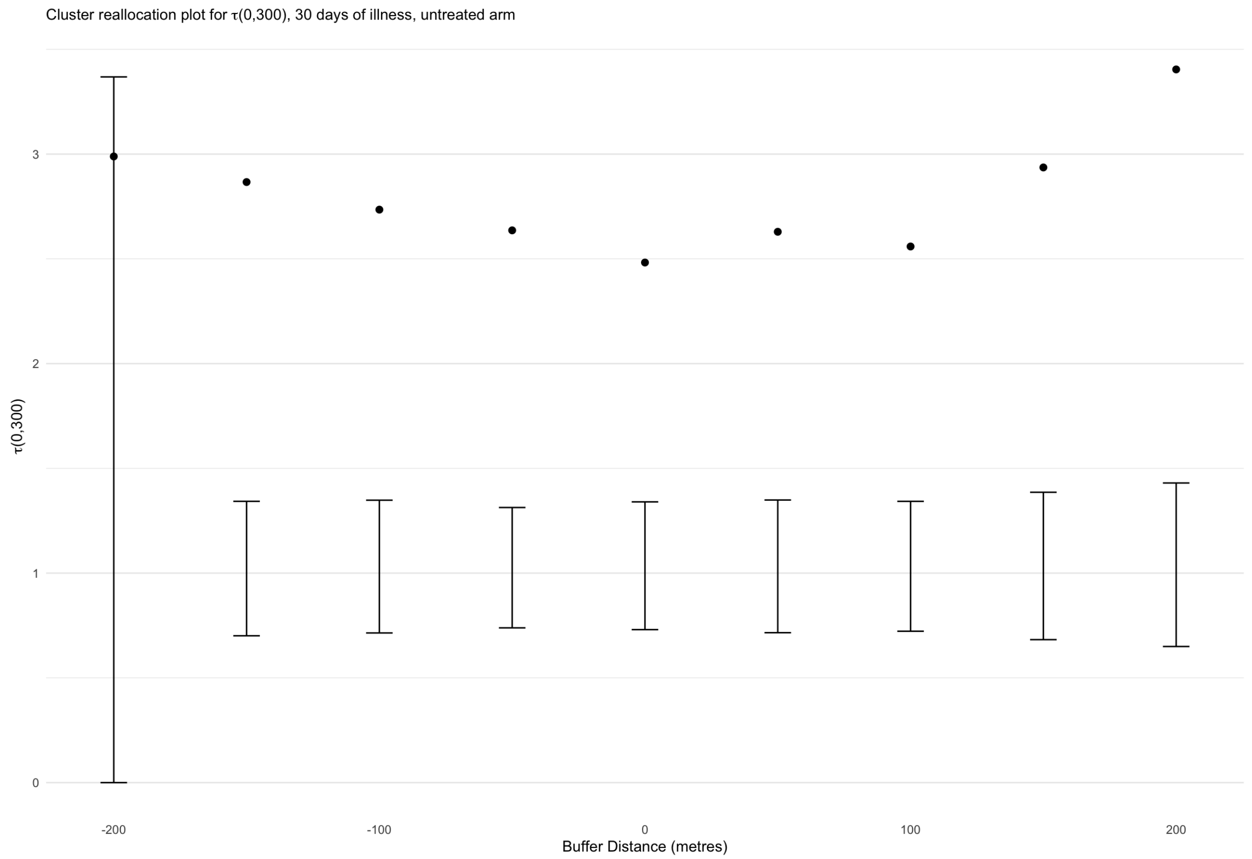

Figure S1: The spatial clustering measure, τ, (with a space-time window of 300m and 30d) in control clusters in the AWED trial, after cluster reallocation at various buffer distance.s

	**Aggregate**			**Intervention**			**Control**		
Exposure	DENV infection	test negative	OR (CI)	DENV infection	test negative	OR (CI)	DENV infection	test negative	OR (CI)
Exposed	42	64	11.2	3	13	10.2	39	51	8.31
Unexposed	343	5857	(6.21, 20.2)	64	2825	(1.90, 54.7)	279	3032	(4.47, 15.5)
Variable		OR		(95%CI)		p-value			
Exposure		8.31		(4.47, 15.5)		0.00	<0.001		
Intervention		0.246		(0.17, 0.36)		0.00	<0.001		
Exposure*Intervention		1.23		(0.20, 7.35)		0.82			

Table S1: Effects of intervention and exposure on infection with dengue (DENV), where a participant's exposure is defined by a dengue infection occurrence (of another participant) within the prior 7 days and with a home location within 100 metres of the participant.

Serotype	Exposure	Aggregate			Intervention			Control		
		DENV1	test negative	OR (CI)	DENV1	test negative	OR (CI)	DENV1	test negative	OR (CI)
DENV1	Exposed	2	8	26.9	0	3	0.00	2	5	28.6
	Unexposed	55	5913	(5.55, 130)	12	2835	-	43	3078	(5.35, 153)
DENV2	Exposed	5	33	17.2	1	3	85.9	4	30	9.93
	Unexposed	52	5888	(6.55, 44.9)	11	2835	(8.29, 891)	41	3053	(3.42, 28.8)
DENV3	Exposed	0	7	0.00	0	1	0.00	0	6	0.00
	Unexposed	57	5914	-	12	2837	-	45	3077	-
DENV4	Exposed	2	14	15.3	0	2	0.00	2	12	11.9
	Unexposed	55	5907	(3.41, 69.1)	12	2836	-	43	3071	(2.54, 55.7)

Table S2: Effects of intervention and exposure on infection with dengue serotype DENV1, where a participant's exposure is defined by a dengue infection occurrence (of another participant) within the prior 7 days and with a home location within 100 metres of the participant.

Serotype	Exposure	Aggregate			Intervention			Control		
		DENV2	test negative	OR (CI)	DENV2	test negative	OR (CI)	DENV2	test negative	OR (CI)
DENV1	Exposed	5	8	24.8	2	3	105	3	5	14.1
	Unexposed	149	5913	(8.76, 70.2)	18	2835	(16.5, 666)	131	3078	(4.05, 49.1)
DENV2	Exposed	18	33	23.6	0	3	0.00	18	30	15.8
	Unexposed	136	5888	(12.3, 45.3)	20	2835	-	116	3053	(8.10, 30.8)
DENV3	Exposed	0	7	0.00	0	1	0.00	0	6	0.00
	Unexposed	154	5914	-	20	2837	-	134	3077	-
DENV4	Exposed	4	14	11.3	0	2	0.00	4	12	7.87
	Unexposed	150	5907	(3.03, 41.7)	20	2836	-	130	3071	(2.04, 30.3)

Table S3: Effects of intervention and exposure on infection with dengue serotype DENV2, where a participant's exposure is defined by a dengue infection occurrence (of another participant) within the prior 7 days and with a home location within 100 metres of the participant.

Serotype	Exposure	Aggregate			Intervention			Control		
		DENV3	test negative	OR (CI)	DENV3	test negative	OR (CI)	DENV3	test negative	OR (CI)
DENV1	Exposed	0	8	0.00	0	3	0.00	0	5	0.00
	Unexposed	27	5913	-	5	2835	-	22	3078	-
DENV2	Exposed	0	33	0.00	0	3	0.00	0	30	0.00
	Unexposed	27	5888	-	5	2835	-	22	3053	-
DENV3	Exposed	5	7	192	2	1	1891	3	6	81.0
	Unexposed	22	5914	(54.6, 675)	3	2837	(94.7,37771)	19	3077	(22.0, 298)
DENV4	Exposed	2	14	33.8	0	2	0.00	2	12	25.6
	Unexposed	25	5907	(7.96, 143)	5	2836	-	20	3071	(5.98, 109)

Table S4: Effects of intervention and exposure on infection with dengue serotype DENV3, where a participant's exposure is defined by a dengue infection occurrence (of another participant) within the prior 7 days and with a home location within 100 metres of the participant.

		Aggregate			Intervention			Control		
Serotype	Exposure	DENV4	test negative	OR (CI)	DENV4	test negative	OR (CI)	DENV4	test negative	OR (CI)
DENV1	Exposed	2	8	17.4	0	3	0.00	2	5	18.1
	Unexposed	85	5913	(3.64, 83.1)	17	2835	-	68	3078	(3.45, 95.0)
DENV2	Exposed	1	33	2.07	0	3	0.00	1	30	1.47
	Unexposed	86	5888	(0.279, 15.4)	17	2835	-	69	3053	(0.197, 11.0)
DENV3	Exposed	0	7	0.00	0	1	0.00	0	6	0.00
	Unexposed	87	5914	-	17	2837	-	70	3077	(0.00, 0.00)
DENV4	Exposed	4	14	20.3	0	2	0.00	4	12	15.5
	Unexposed	83	5907	(6.51, 63.5)	17	2836	-	66	3071	(4.79, 50.2)

Table S5: Effects of intervention and exposure on infection with dengue serotype DENV4, where a participant's exposure is defined by a dengue infection occurrence (of another participant) within the prior 7 days and with a home location within 100 metres of the participant.
